# ITS-NANO - Prioritising nanosafety research to develop a stakeholder driven intelligent testing strategy

**DOI:** 10.1186/1743-8977-11-9

**Published:** 2014-02-13

**Authors:** Vicki Stone, Stefano Pozzi-Mucelli, Lang Tran, Karin Aschberger, Stefania Sabella, Ulla Vogel, Craig Poland, Dominique Balharry, Teresa Fernandes, Stefania Gottardo, Steven Hankin, Mark GJ Hartl, Nanna Hartmann, Danial Hristozov, Kerstin Hund-Rinke, Helinor Johnston, Antonio Marcomini, Oliver Panzer, Davide Roncato, Anne T Saber, Håkan Wallin, Janeck J Scott-Fordsmand

**Affiliations:** 1 Heriot Watt University, City of Edinburgh, UK; 2 Veneto Nanotech, Padova, Italy; 3 Institute of Occupational Medicine, Midlothian, UK; 4 European Commission, DG-JRC, Ispra, Italy; 5 Italian Institute of Technology, Center for Bio-Molecular Nanotechnologies @ UniLe, Via Barsanti - 73010, Arnesano (Lecce), Italy; 6 National Research Centre for the Working Environment, Copenhagen, Denmark; 7 Fraunhofer IME, DE, Aachen, Germany; 8 European Research Services GmbH, DE, Edinburgh, UK; 9 Centro Ricerche Fiat S.C.p.A, IT, Bedfordshire, UK; 10 Aarhus University, Aarhus, Denmark; 11 Technical University of Denmark, Kongens Lyngby, Denmark

**Keywords:** Nanomaterial, Exposure, Hazard, Physicochemical, Grouping, Ranking, Modelling, Risk assessment, High throughput, Mode-of-action

## Abstract

**Background:**

To assess the risk of all nanomaterials (NMs) on a case-by-case basis is challenging in terms of financial, ethical and time resources. Instead a more intelligent approach to knowledge gain and risk assessment is required.

**Methods:**

A framework of future research priorities was developed from the accorded opinion of experts covering all major stake holder groups (government, industry, academia, funders and NGOs). It recognises and stresses the major topics of physicochemical characterisation, exposure identification, hazard identification and modelling approaches as key components of the current and future risk assessment of NMs.

**Results:**

The framework for future research has been developed from the opinions of over 80 stakeholders, that describes the research priorities for effective development of an intelligent testing strategy (ITS) to allow risk evaluation of NMs. In this context, an ITS is a process that allows the risks of NMs to be assessed accurately, effectively and efficiently, thereby reducing the need to test NMs on a case-by-case basis.

For each of the major topics of physicochemical characterisation, exposure identification, hazard identification and modelling, key-priority research areas are described via a series of stepping stones, or hexagon diagrams structured into a time perspective. Importantly, this framework is flexible, allowing individual stakeholders to identify where their own activities and expertise are positioned within the prioritisation pathway and furthermore to identify how they can effectively contribute and structure their work accordingly. In other words, the prioritisation hexagon diagrams provide a tool that individual stakeholders can adapt to meet their own particular needs and to deliver an ITS for NMs risk assessment. Such an approach would, over time, reduce the need for testing by increasing the reliability and sophistication of *in silico* approaches.

The manuscript includes an appraisal of how this framework relates to the current risk assessment approaches and how future risk assessment could adapt to accommodate these new approaches. A full report is available in electronic format (pdf) at http://www.nano.hw.ac.uk/research-projects/itsnano.html.

**Conclusion:**

ITS-NANO has delivered a detailed, stakeholder driven and flexible research prioritisation (or strategy) tool, which identifies specific research needs, suggests connections between areas, and frames this in a time-perspective.

## Background

Prioritisation of research activities and funding is a perpetual issue, especially when it comes to applied research, *i.e.* research that directly affects society. With respect to Nanotechnology, the development and adaptation of methods to assess the safety of nanomaterials (NMs) are currently under pressure as NMs are being made and developed in increasing types and quantities [1]. Stakeholders, including academics, industry, regulators and NGOs therefore require a streamlined process, known as an intelligent testing strategy (ITS) that allows the risks of NMs to be performed accurately, effectively and efficiently [2-4]. Accurately means that the correct conclusion regarding risk is made, while effectively means that appropriate tools/protocols are available to achieve the risk assessment, and efficiently means that the assessment does not take too long or cost too much money.

In 2012 the European Commission funded a project, ITS-NANO, to prioritise research that would allow development of an ITS for NMs safety (http://www.nano.hw.ac.uk/research-projects/itsnano.html). The ambition was to develop research prioritisation that would be adopted and recognised by all relevant stakeholders. The ITS-NANO consortium included experts from nine different European organisations (see the author details), who engaged with over 80 expert representatives from academia, industry, regulators, funders and NGOs. Interaction was facilitated via two workshops to assess the stakeholder needs, to identify and confirm gaps in knowledge and, based upon these different sources of information, to deliver a stakeholder driven research prioritisation document.

The following manuscript outlines the approach taken to assess gaps in knowledge, to identify the research required to fill these gaps, and finally how to prioritise these gaps according to the needs and opinions of a broad spectrum of stakeholders. In doing so ITS-NANO provides a tool to organise and prioritise research activities that will lead to an ITS which will be fit for purpose and develops with time.

## Results and discussion

To assess the risk of every NMs on a case-by-case basis for every possible human and environmental exposure scenario [1] is impossible. Instead a more intelligent approach to knowledge gain and risk assessment is required [2-4]. Currently we lack the knowledge required to accurately predict the risks of NMs using either empirical testing or modelling approaches. Therefore research is required that will drive the field forward in a focused way that will deliver such an intelligent approach.

### Gap analysis

**Figure 1:**
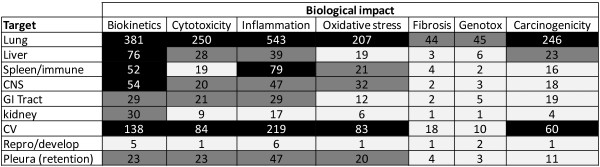
**A heat map illustrating the number and pattern of nanomaterial publications identified (December 2012) in Web of Science and PubMed.** This particular example focuses on systemic effects identified in human toxicity studies using both *in vitro* and *in vivo* approaches. Black signifies more than 50 publications, grey represents 20–50 publications while white is less than 20. A full set of heat maps for local and systemic effects for human toxicity as well as for ecotoxicity is provided in the gap analysis at http://www.nano.hw.ac.uk/research-projects/itsnano.html.

A gap analysis of the available knowledge required to assess the risks of NMs and to develop an intelligent testing strategy was conducted. The gaps covered physicochemical characterisation, exposure assessment, hazard assessment, grouping/ranking/modelling approaches and risk assessment methodologies/frameworks. Owing to the large number of publications pertaining to hazard, these were represented and analysed via a series of heat map tables (Figure 1). The heat maps proved to be a novel and extremely useful tool for visually identifying where gaps in our knowledge of NMs safety exist [5].

### The risk assessment paradigm

The traditional risk assessment paradigm used for chemicals includes assessment of hazard and exposure, by taking into account physicochemical information. The ITS presented here also uses the same risk assessment approach, but it **increases the emphasis on thorough physicochemical characterisation of NMs** compared to the approach currently used. This means that the risk assessment paradigm was adapted, where necessary, to take account of NM-specific or NM-relevant factors, such as size, shape and surface characteristics. All three aspects (physicochemical, exposure and hazard) combined with cross-cutting grouping/ranking were defined and used to identify the research needed to deliver the tools required for robust risk assessment of NMs.

### Defining the ITS-NANO vision and time frame

Based upon the opinions of the project partners with input from the stakeholders workshop (Edinburgh, 2012), the ITS-NANO vision was identified as a way forward in which ‘***there is a knowledge-based sustainable development of engineered NMs, that is based upon robust procedures for effective management of the risks of existing and future NMs***’ [6].

In order to identify the research priorities to achieve the ITS, the actual ITS ambition itself was outlined. In the short term (less than 5 years) this includes improving the understanding of the relationships between physicochemical, exposure and hazard characteristics (e.g. by determining the mode-of-action underlying the hazardous effects), primarily to promote the development of grouping and or ranking approaches for NMs and to enable design of *in vitro* and high through-put screening tools that target biological key processes. Such approaches are required to improve the efficiency of NM screening and risk assessment. In the mid-term (5–10 years) the ambition includes an understanding of the relevance to risk assessment of less demanding, costly and time consuming approaches (e.g. high throughput (HTP) systems that analyse large numbers of samples simultaneously and *in vitro* models) compared with more traditionally used techniques, in order to develop a faster evaluation of risk. In the longer-term (10–15 years) risk assessment will require the development of increasingly robust modelling approaches to allow a reduced requirement for *in vivo* and *in vitro* hazard testing, while in the distant future (>15 years) risk assessment will be increasingly reliant on modelling/*in silico* approaches, with focused physicochemical, exposure and hazard testing only if additional information is required.

### Defining the ITS components

For each element of the risk assessment (physicochemical, exposure and hazard) the essential information required was defined to generate an Identity (ID) [6].

The Physicochemical ID was defined as ‘***the dynamic pattern of physical and chemical characteristics (identified using appropriate analytical techniques) associated with one or several specified NMs during their life cycle****’*. This includes identifying detailed physicochemical descriptors of key inherent features of the NMs in terms of what they are (composition, size etc.), where NMs go (biological and environmental fate) and what they do (inherent activity of NMs).

The Exposure ID was defined *as ‘****the pattern of concentrations of one or more NMs in different matrices (air, liquid or solid) and as a function of duration and variability over time during their life cycle****’*. This takes into account both human and environmental exposure routes. In risk assessment the Exposure ID is critical for linking the Physicochemical ID to the Hazard ID.

A Hazard ID was defined as ‘***the pattern of biological responses (determined using appropriate combinations of toxicological and ecotoxicological models, tests and endpoints) associated with one or several specified NMs****’*. Human and environmental Hazard IDs were integrated in order to promote collaboration and knowledge exchange between the two disciplines. This is important since it is likely that many similarities exist between the modes-of-action of NMs underlying toxicity, and therefore techniques can be shared to study hazard impacts.

Grouping was defined as ‘***the arrangement of nanomaterials into groups based on common attributes***’. In the context of risk assessment, this could include a common hazardous physicochemical property, or an exposure potential that infers greater risk. Ranking was defined as ‘***assigning a position in a scale***’, meaning that, NMs may be classified based on attributes describing their potential for exposure (e.g. high dustiness) and/or their high intrinsic toxicity. Whereas ranking does not necessarily imply a relationship between the NMs on a given scale, grouping does infer a relationship in a common attribute. It is worth noting that groups can be ranked and ranking can occur within groups.

The risk assessment (RA) framework in the context of this document was defined as “**the entirety of EU law requiring a RA of substances for their safe use as such or in products/articles and the related guidance**”. The RA framework is therefore considered to be applicable to NMs, even if they are not always explicitly addressed. However, some adaptations to risk assessment approaches and regulations may be required to ensure the safety of current and future NMs and their applications, as well as the integration of new tools (e.g. quantitative nano-structure activity relationships [7,8], or multi-component decision analysis [9]) used to assess risk.

### Prioritising the research needs for the ITS components

The research priorities identified in the project, including future time-frames, are presented below. Note that the longer term priorities are not considered less important, rather that they will require more information to be addressed before they are satisfied. Therefore work on the longer term goals needs to start now in order to appropriately frame the short-term research required. This will ensure that the outputs from the short-term priorities are appropriate for development of the longer term priorities. The following sections summarise research priorities divided according to the elements of the risk assessment, as well as grouping/ranking and implementation of the findings into risk assessment frameworks [6].

(i) Physicochemical ID

**Figure 2:**
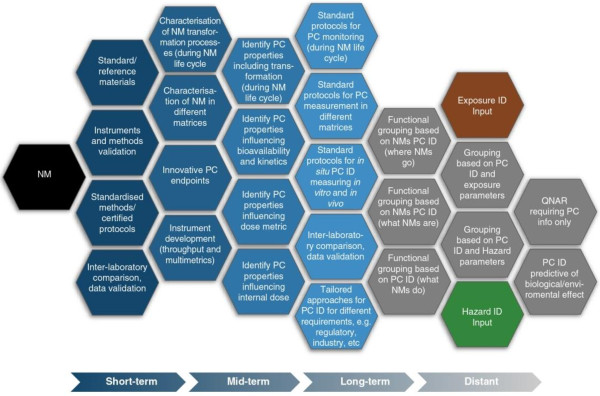
**Proposed research prioritisation for generating an effective PC ID to inform an Intelligent Testing Strategy.** The research priorities are graded across the diagram, with hexagons to the left being of short term-priority (< 5 years) stretching to longer term priorities on the right (> 15 years). Grey hexagons represent modelling components that will lead to the ITS. The short-term priorities should be considered in the context of the long-term priorities to ensure that they generate the information needed to provide robust foundations for the longer-term priorities.

The Physicochemical ID refers to a set of characteristics, which are likely to change over the life cycle of a NM, and that can potentially be used for risk assessment and decision-making purposes. In the short term, tools are required that include standard/reference materials, validated instruments and standard protocols to maximise the cost-effectiveness of physicochemical characterisation. A library of such tools is needed, so that stakeholders can tailor the selection of tools to their own personal requirements. In the mid-term these tools will contribute to effective characterisation of materials at different life cycle stages and in a wide array of complex matrices (e.g. in products such as composite materials, plastics and food, but also in different environments such as water, air or soil). Stakeholders already employ a wide array of physicochemical characteristics, but the development, validation and implementation of novel nano-relevant physicochemical descriptors, techniques and instrumentation may be required. In the long-term, stakeholders will require further development of the standardised protocols for detecting, monitoring and characterising NMs throughout their life cycle, in complex matrices and for both *in vitro* and *in vivo* models. Development of this expanded set of tools will allow the approach to physicochemical ID to be flexible, tailored and/or tiered. Finally, in the distant future, high-quality physicochemical data for *in vitro, in vivo* and *in silico* approaches will be required to support exposure assessment and hazard identification (Figure 2).

(ii) Exposure ID

**Figure 3:**
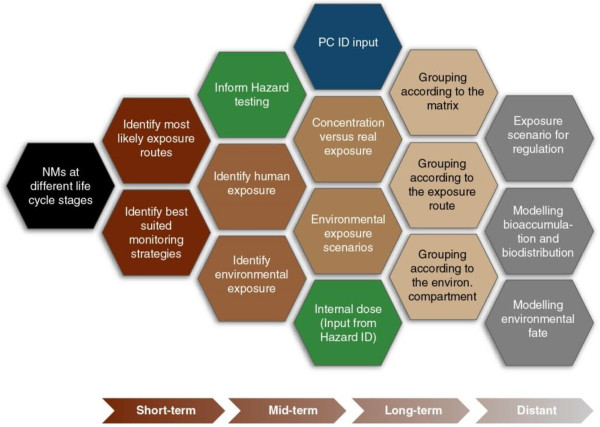
**Proposed sequence of events for implementing an exposure testing strategy aimed at grouping and modelling NMs.** The research priorities are graded across the diagram, with hexagons to the left being of short term-priority (< 5 years) stretching to longer term priorities on the right (> 15 years). Grey hexagons represent modelling components that will lead to the ITS. The short-term priorities should be considered in the context of the long-term priorities to ensure that they generate the information needed to provide robust foundations for the longer-term priorities.

As for the Physicochemical ID, Exposure ID also requires the standardisation of methods for discriminating NMs from background particles in complex matrices, throughout their life cycles. This research need should be retained as a continuous priority over time. In the short term, for assessing human exposure, research priorities include both inhalation and ingestion routes. The inhalation research needs to reflect the potential for exposure to workers in the occupational settings, while ingestion research reflects both intentional and incidental exposure to NMs in food and consumer products or at workplaces due to poor hygiene; dermal research reflects potential for exposure of consumers using cosmetic products or other consumer products and of workers in occupational settings. In parallel, exposure assessment needs to better define the relationship between exposure concentration and internal dose. For assessing environmental exposure, the research focus needs to include identification of long-term accumulation as well as concentration hotspots in both soils and sediments. In the mid-term, actual exposure concentrations in the matrices of different environmental compartments (e.g. air, water, soil) should be linked to actual exposure. In addition, robust strategies for sampling and determining concentrations in appropriate indicator organisms and/or potentially sensitive environmental compartments need to be formulated and thoroughly validated. To accelerate physicochemical characterisation, high throughput (HTP) screening will be essential, while modelling approaches will be required to reduce the burden of testing. This approach will facilitate grouping of NMs and modelling their exposure, bioaccumulation and fate throughout their life cycles. In the distant future the development of standardised protocols for multi-metric and innovative detection tools is essential (Figure 3).

(iii) Hazard ID

**Figure 4:**
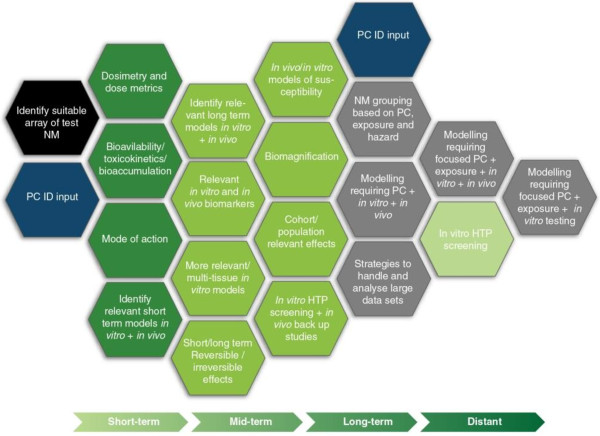
**The research steps required to formulate a Hazard ID for incorporation into the ITS.** The research priorities are graded across the diagram, with hexagons to the left being of short term-priority (< 5 years) stretching to longer term and distant priorities on the right (> 15 years). Grey hexagons represent modelling components that will lead to the ITS. The short-term priorities should be considered in the context of the long-term priorities to ensure that they generate the information needed to provide robust foundations for the longer-term priorities.

Hazard ID generation requires that *in vitro* and *in vivo* models are used to assess the local and systemic effects of NMs (acute and chronic) and that the mode-of-action of NMs is identified to better understand what responses can be used to screen NM toxicity. For Hazard ID generation, key short-term priorities are to develop dose metrics that allow determination of the toxicokinetics, bioavailability and mode of action of NMs. In the mid-term, appropriate validated *in vitro* and *in vivo* models need to be developed to predict long-term or chronic effects. These models will also require the development of reliable biomarkers to estimate exposure and/or establish indicators for chronic effects. *In vivo* models will allow determination of time courses of responses including distinction between short and long term effects, rapid and delayed onset, reversible and irreversible effects, and underlying mode-of-action. However, the long term goal is to develop and validate alternative non-animal models to replace such *in vivo* models based on the identification of key biological processes that drive the adverse effects. In addition to validation of simple culture systems, there will be a need to generate more relevant multi-cell and multi-tissue (e.g. gut, endothelium and liver) *in vitro* models. In the long-term, knowledge of the population-level effects, bioaccumulation and biomagnification of NMs will be required. A common approach linking mammalian toxicology and ecotoxicology studies is encouraged. Studies will generally require robust, appropriate *in vitro* and *in vivo* models of susceptibility to focus on vulnerable individuals or populations. Identification of the mode-of-action of hazardous effects will allow design of *in vitro* models targeting key and relevant processes rather than an indirect indicator or something that is simply easy to measure. In the distant future *in vitro* HTP testing and *in silico* models will allow focused hazard assessment with an eventual reduction in the burden of testing (Figure 4).

(iv) Grouping, Ranking and Modelling approaches

**Figure 5:**
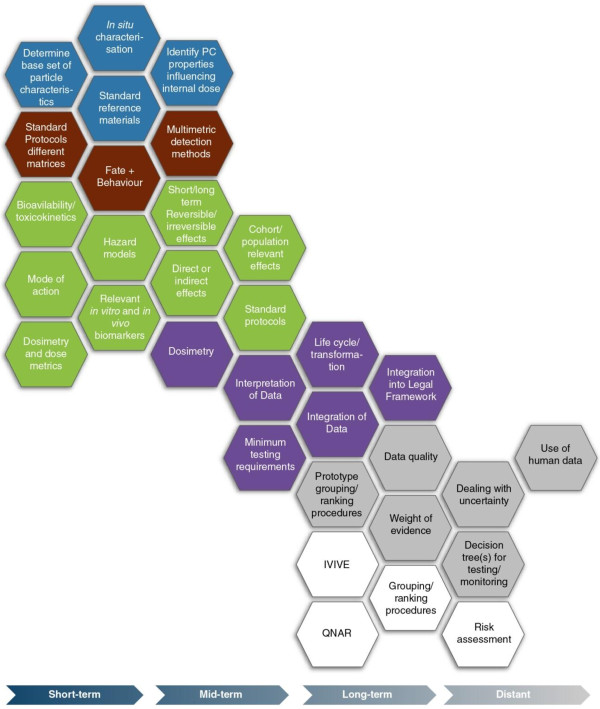
**The diagram identifies the components required for the development of a grouping/ranking approach for NMs.** Hexagon colours relate to PC ID (blue), Exposure (brown), Hazard (green), Cross-cutting issues, implementation into a RA framework (grey) and the final goal of the ITS (white). The diagram is intended to start on the left (NM) and finish on the right, but there is no strict order of passage between the hexagons to achieve the final goal. The research priorities are graded across the diagram, with hexagons to the left being of short term-priority (< 5 years) stretching to longer term and distant priorities on the right (> 15 years). It is important to note that contrary to similar representations in preceding chapters, the hexagons for grouping/ranking are not necessarily intrinsically linked, but contribute to overall progress towards grouping and/or ranking of NMs as well as modelling. This example is dominated by hazard, but in other scenarios the exposure or physicochemical priorities may be more dominant.

Informative grouping/ranking requires precise and accurate Physicochemical-, Hazard-, and Exposure-ID inputs. Such data can be used to group or rank materials e.g. using weight of evidence approaches or structural activity relationships, which will highly enhance cross material information flow. In particular, the mode-of-action of NMs and how this relates to a defined set of physicochemical characteristics is also crucial in development of grouping, ranking or modelling tools. It is envisaged that for NMs new approaches should be developed for grouping, ranking and extrapolation/interpolation of results between species/models and between materials (Figure 5).

#### Common themes

Specific, detailed recommendations for each aspect of the strategy are presented under each heading, but as emphasised, an integrative research effort is required.

Key cross-cutting issues were identified including the development of standard protocols, reference or standard materials, and easily adaptable HTP techniques. This approach will lead to the generation of libraries of standard protocols to allow a tailored or streamlined approach to testing. Since this library is likely to be quite extensive, it will be necessary to support it with a decision tree or matrix to allow individual stakeholders to identify the protocols most relevant to them. Different standard materials may be required for different applications (e.g. for both calibration of a microscope and toxicity testing), it would also be desirable to streamline the range of potential reference and standard materials so that, where possible, the same material can be used for multiple applications.

Another cross-cutting issue involves the generation of HTP techniques for all aspects of NM testing. HTP techniques are equally relevant to physicochemical, exposure and hazard scenarios. Where possible it will also be advantageous to make HTP approaches multi-metric, allowing multiple different endpoints to be assessed in a single system, e.g. a single HTP system could measure PC characteristics alongside hazard endpoints, or hazard alongside exposure.

Cross-cutting issues also include the development and implementation of: (i) a common language (i.e. shared ontology, terminology and nomenclature); (ii) comprehensive, user-friendly information-sharing tools (e.g. databases); (iii) synergistically applicable advanced techniques (by providing, for instance, an efficient research framework and facilitating access to advance analytical equipment and qualified, highly trained staff); and (iv) in-depth risk assessment methodologies.

### Implementation of the ITS into risk assessment and regulation

Successful application of the ITS-NANO research prioritisation will lead to the generation of relevant information on NM physicochemical characteristics, hazards and exposure, including data obtained from *in vitro* tests, read-across/grouping/ranking, and *in silico* hazard and exposure models. Thus, these research outputs will provide secure, evidence-based foundations for formulating and implementing ‘best practices’ for risk assessment and for data management of NMs [6].

**Figure 6:**
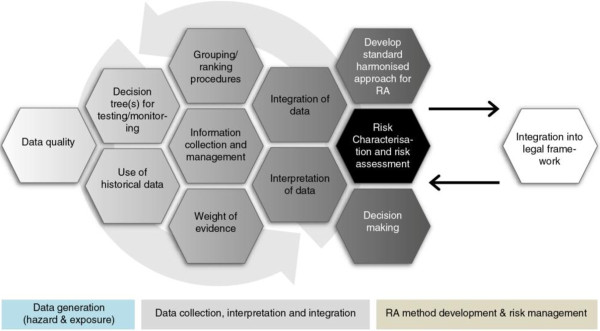
**An overview of the risk assessment of NMs in the context of the ITS-NANO research strategy.** The grey arrows indicate an iterative process and the boxes below represent the steps of data generation (for both hazard and exposure data), data collection, interpretation and integration as well as risk assessment method development and risk management.

Within existing risk assessment frameworks, alternative to animal testing (e.g. *in vitro*) and non-testing (e.g. read-across) methods are already encouraged, provided they are validated or scientifically justified [10,11]. However, mid- to long-term issues are foreseen, including the potential need to adapt the current regulatory framework to accommodate the novel quantitative tools and probabilistic approaches to integrate data from alternative approaches into a risk assessment. To facilitate this process, training of risk assessors, regulators and researchers will be required, along with additional guidance for interpretation and integration of these data and their regulatory acceptance (Figure 6).

### Relationship to parallel strategy activities

A number of other reports address the requirements for future research in the area of nanomaterial risk assessment. Each report is very insightful and supports the outcomes of ITS-NANO. For example, the National Research Council (USA) published a report entitled Toxicity Testing in the 21st Century: A Vision and Strategy [2]. The report addresses human toxicity testing rather than risk assessment and it focuses on chemicals in general, rather than NMs. Similar to ITS-NANO the report predicts expanded use of high- and medium-throughput *in vitro* screening assays as well as computational toxicology with reduced animal testing. This is similar to the ambition proposed by ITS-NANO, however, the options presented do not develop over time.

Recently, a multi-stakeholder perspective on the use of alternative testing strategies for assessing NM safety has been published [3] which stresses the need to move towards reduced animal testing and an increased reliance on *in vitro* HTP testing, high content screening and *in silico* approaches. Based on a carbon nanotube example, a tiered approach to testing was proposed in which predictive modelling and *in vitro* models would be used to prioritise the carbon nanotubes to be used for short term inhalation or instillation experiments with rodents, which would then be used to design subsequent longer term (90 day exposure) studies. This approach is based upon current capabilities and fits nicely into the framework provided by ITS-NANO for development, streamlining and improvement in the future.

A strategic research agenda for nanosafety, covering 2015–2025, has recently been published by the NanoSafety Cluster (European Commission FP7 funded research projects) [4]. This document provides an overview of all research needs related to human and environmental nanosafety. A recommendation for the development of an ITS is included within this document, which provides a wider context and framework in which the detailed ITS-NANO strategy fits, where it can be related to other wider activities.

## Conclusions

**Figure 7:**
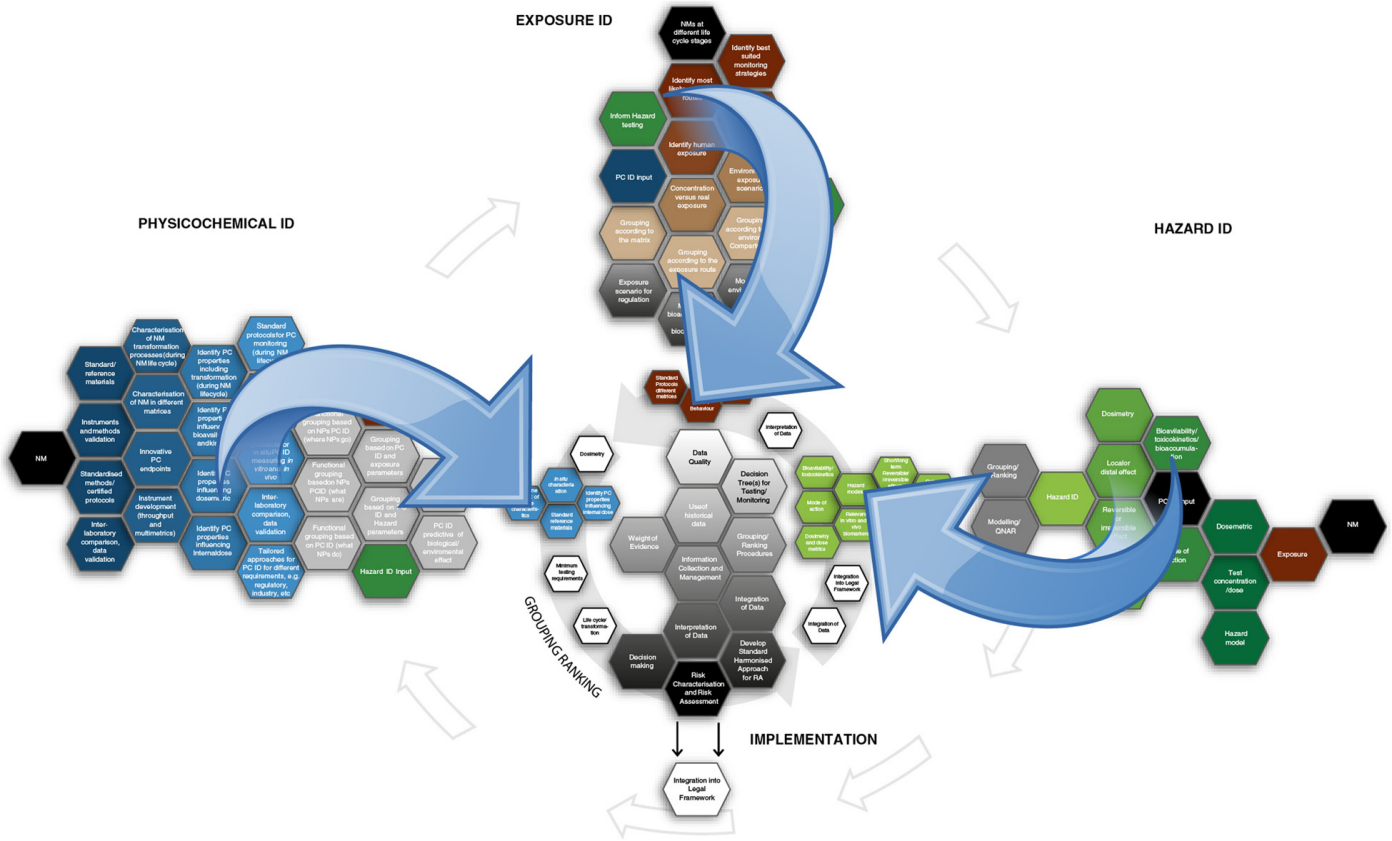
**The diagram illustrates the connections between the identified research priorities, and the implementation of the subsequent acquired knowledge and methods in the risk evaluation process.** Each hexagon represents a priority research need, and each interface a logical relationship; with black hexagons representing NMs around the outside and the ITS modelling tools in the centre. Between the three priority research areas (Physicochemical, Exposure and Hazard ID) and the central ITS are the grouping/ranking approaches (bold hexagons) needed to streamline the data requirements. The blue arrows indicate the direction of research progress over time (from the perimeter of the diagram towards the core). The outputs of the ITS feed into the risk assessment frameworks at the bottom of the diagram.

ITS-NANO has delivered a detailed, stakeholder driven and flexible research prioritisation (or strategy) tool, which identifies specific research needs, suggests connections between areas, and frames this in a time-perspective. If implemented this research prioritisation programme would lead to the development of an ITS for NMs. The strategy is provided in the form of text and diagrams in order to appeal to, and be useable by a wide audience. The individual diagrams from each aspect of the paper can be combined to provide an overall strategy diagram (Figure 7) that demonstrates how the elements link together and evolve over time.

The clear and flexible nature of the summary diagrams allows all stakeholders to identify the key research questions and priorities that are relevant to their needs and provides a framework in which to structure and integrate these activities. The full ITS-NANO report goes even further to provide detailed outlines of how each research priority could be addressed. The flexible nature of the diagrams also allows them to evolve over time as individual research priorities are addressed and new knowledge is acquired.

The usefulness and success of this framework is obviously dependent on whether it is employed and used strategically in, for example, calls for research, or implementation in risk assessment procedures. In fact, following communication of the ITS-NANO research prioritisation findings during a webinar to over 70 stakeholders (May 2013; available online: http://www.nano.hw.ac.uk/research-projects/itsnano.html), the approach used by ITS-NANO has already been employed in other European Commission funded projects (e.g. MARINA) and the outputs are being integrated into new projects (e.g. NANoREG).

Finally, it must be emphasised that in order to ensure a fast and sustainable development of NMs it is up to the research community and the other stakeholders to ensure that their nanosafety-related activities are directed into a framework, such as the above. A full report is available in electronic format (pdf) at http://www.nano.hw.ac.uk/research-projects/itsnano.html.

## Methods

### Gap analysis

A literature search of Web of Science and Pubmed was conducted using systematically identified keywords to cover all relevant aspects of research required for risk assessment of NMs. These key words included different descriptors to encompass nanomaterials (e.g. nanoparticle, nanomaterial, nanotube, nanowire, nanorod etc.), combined with key words relevant to either exposure (e.g. exposure, uptake, inhalation, ingestion, airborne), hazard (e.g. cytotoxicity, toxicity, lethality) or physicochemical characterisation (e.g. size, surface area, crystallinity). Then heat map tables were generated that described the number of relevant publications identified relating to different target species, routes of exposure, physicochemical characteristics and mode of action. Examples of heat map tables are provided in Figure 1. Full details of the protocol are described in the *Identification of Knowledge Gaps and Strategic Priorities for Human and Environmental Hazard, Exposure and Risk Assessment of Engineered Nanomaterials* document, (http://www.nano.hw.ac.uk/research-projects/itsnano.html).

The output of the literature assessment, including heat maps, was developed into a knowledge gap analysis. On this basis we identified and listed outstanding research, allowing for effective risk assessment of NMs especially in relation to development of an ITS. The gaps were grouped logically according to subject content, but they were not ranked according to priority at this stage. This gap analysis was supported by an online questionnaire distributed to on-going EU nanosafety research projects, in order to estimate the expected knowledge gain within the next few years.

The draft gap analysis was shared with a group of 40 stakeholders representing experts from academia, industry, regulators, funders and NGOs at a two day workshop in Edinburgh (September 2012). The experts were asked to assess whether the gaps identified were appropriate and whether they corresponded to their experience/knowledge and/or whether any gaps had been missed. Useful feedback was provided to allow a more accurate representation of current knowledge gaps. The final gap analysis is available on-line (http://www.nano.hw.ac.uk/research-projects/itsnano.html).

### Structuring the risk assessment paradigm

Based upon the gap analysis, the ITS-NANO team drafted initial ideas for research prioritisation. It was recognised that the future risk assessment paradigm for NMs would follow the structure of the framework currently used for chemicals, in that it would include an assessment of hazard and exposure. However, the prioritisation document also discussed how to better incorporate physicochemical characterisation of NMs into the risk assessment process. For this reason, during the Edinburgh workshop, the experts were asked to identify what is necessary to define a hazard identity (ID), exposure ID and physicochemical ID for NMs. In addition, ideas were invited with respect to the development of grouping, ranking and modelling approaches for streamlining testing and implementation into risk assessment frameworks.

### Prioritising the identified research needs

Based upon discussions between the project partners, along with input from the Edinburgh workshop stakeholders, the vision and ambitions were identified.

Once the research needs were identified and ordered with respect to the ITS-NANO vision, a series of draft hexagon diagrams for hazard identity (ID), exposure ID and physicochemical ID, as well as for grouping, ranking and modelling approaches and implementation into risk assessment frameworks were generated. These included two types of diagram, the first outlined the information required to generate each ID, grouping/ranking or implementation of the research outputs into a risk assessment framework. The second series of diagrams outlined the research required to achieve each ID, grouping/ranking or implementation. A second stakeholder workshop (Venice, March 2013) was then conducted in which 80 stakeholders were asked to comment on the content of the draft diagrams. First they refined the information required to generate each ID and then they prioritised the research needs. Following the workshop each diagram was adapted to take into account the stakeholder feedback, resulting in a clear prioritisation of the research needs.

Concurrent with this prioritisation it was investigated how such development could be implemented in the current and future risk assessment regulations.

## Abbreviations

ID: Identity; HTP: High throughput; RA: Risk assessment.

## Competing interests

Vicki Stone currently receives research funding from GlaxoSmithKline, and in the past she has received research funding from Unilever. VS collaborates (but does not receive funding from) a variety of NM producers (e.g. BASF and Nanocyl) via the FP7 project SUN. Mark Hartl has no industry funding in this area. Janeck J. Scott-Fordsmand collaborates (but does not receive funding from) a variety of NM producers (e.g. BASF and Nanocyl) via various FP7 projects. NRCWE partners (AT Saber , H Wallin, U Vogel) declare no industrial funding in this area but collaborations with industrial NM producers and users in the EU projects NanoReg, NanoMile and Gladiator.

## Authors’ contributions

VS Project coordinator, Work Package leader for assessing the State of the Art, Author of Hazard element of ITS-NANO report, editor of full report, lead author. SPM, VN, Italy. Lead author for Exposure ID and Physicochemical ID chapters. Lead organizer for Stakeholder engagement. LT, CP, SH, Institute of Occupational Medicine, UK. Lead authors of Grouping and Ranking Sections. KA, SG, NBH, JRC, Italy. Lead authors of Implementation into Risk Assessment Regulatory frameworks Sections. UV, ATS, HW, NRCWE, Denmark. Lead authors for Hazard Section. DB, TF, MGJH. H. Johnston, Heriot-Watt University, U.K. Authors for assessing the state of the art and for the hazard chapter. SS, IIT, Italy. Lead author for Physicochemical ID Sections. DH, AM, University of Venice, Italy. Authors for Grouping and Ranking. KHR, Fraunhoffer, Germany. Authors for hazard ID Sections. OP, European Research Services, Germany. Overall editing and project management. DR, CRF, Italy. Industry stakeholder input. JJSF, Work package Leader for Research Prioritisation report. All authors read and approved the final manuscript.
